# Inpatient rehabilitation for adult patients with Marfan syndrome: an observational pilot study

**DOI:** 10.1186/s13023-017-0679-0

**Published:** 2017-07-12

**Authors:** Dieter Benninghoven, Denise Hamann, Yskert von Kodolitsch, Meike Rybczynski, Julia Lechinger, Friedrich Schroeder, Marina Vogler, Eike Hoberg

**Affiliations:** 1 Muehlenberg-Clinic for Rehabilitation, Bad Malente-Gremsmühlen, Germany; 2 Clinic of Psychosomatic Medicine and Psychotherapy, University of Luebeck, Lübeck, Germany; 3 Clinic of Cardiology at the University Heart Centre, University of Hamburg, Hamburg, Germany; 4 Marfan Hilfe (Deutschland) e.V. (German Marfan Patient Organization), Eutin, Germany

**Keywords:** Marfan syndrome, Congenital heart defect, Inpatient rehabilitation, Multidisciplinary rehabilitation, Quality of life

## Abstract

**Background:**

Advances in medical, interventional and surgical treatment have increased average life expectancy of patients with congenital heart defects. As a result a new group of adult patients with congenital cardiac defects requires medical rehabilitation. Patients with Marfan syndrome (MFS) are a relevant group among these patients. So far, no reports on the effectiveness of specialized rehabilitation programmes for MFS patients exist. We implemented an inpatient 3-week rehabilitation program for MFS patients at the Muehlenberg-Clinic for rehabilitation and assessed the medical safety as well as the impact of the program on physical fitness and psychological wellbeing of participants by means of an observational pilot study. The comprehensive multidisciplinary program included medical, physiotherapeutic, psychological and social issues. Two groups including 8 and 10 individuals with verified MFS attended the programme. Medically adverse events that occurred during the rehabilitation were registered. Adverse events were defined as: any new cardiac arrhythmias such as atrial fibrillation, ventricular tachycardia, cardiac syncope or any complications located at the aorta. Psychological assessment was performed using Short Form-36 (SF-36), hospital anxiety and depression scale and other psychometric questionnaires. Medical examinations included assessment of maximum power in bicycle ergometry. All assessments were performed at the beginning and at the end of the rehabilitation. Psychometric assessments were repeated 1 year after the end of the programme for both groups, respectively.

**Results:**

Patients were highly satisfied with the programme and improved in almost all psychological and physical fitness assessments. The pre-post-comparison resulted in significant positive changes for mental health (*p* < .001 for SF-36 Mental Health), fatigue (*p* < .05 for Fatigue Severity Scale), nociception (*p* < .05 for SF-36 Pain) and vitality (*p* < .05 for SF-36 Vitality). Physical fitness improved from admission to discharge (*p* < .001 for maximum power in bicycle ergometry, *p* < .05 for maximum nordic walking distance). Considerable improvements persisted through 1 year follow-up. Medical assessments excluded medical problems or adverse events caused by participation in the programme.

**Conclusions:**

In our study, inpatient rehabilitation was both safe and helpful for MFS patients. They benefited in terms of physical fitness, health related quality of life and in terms of psychological wellbeing. An evaluation of the efficacy of the programme in a controlled design as well as further conceptual improvements of our current program is desirable.

## Background

Marfan syndrome (MFS) is an autosomal dominant genetic disorder of the connective tissue. Clinical manifestations are variable and affect different organ systems. Diagnostic criteria are defined by the Ghent criteria [1]. Most serious problems are defects of heart valves and of the aorta. These have caused early death in the past and still give rise to complications without timely treatment. Patients are advised to regularly visit specialized multidisciplinary care centres for medical examination and for particular monitoring of the aorta [2]. Such management strategies allow for careful planning and performing preventive operations with maximum results. Mainly due to improved methods in vascular and cardiac surgery life expectancy of people with MFS has increased, wherefore patients today are facing a normal life expectancy. After surgery or generally in the course of the disease adult patients now demand rehabilitation much more often than in the past. The need for rehabilitation not only relates to cardiovascular problems but to living with MFS in general. A comprehensive and integrated approach to rehabilitation is pivotal. So far, rehabilitation programs specific for MFS appear to exist only in Norway and scientifically evaluated programs do not exist. However sparse studies and case reports emphasize the need for psychosocial support of affected persons [3–5]. All authors agree on the crucial value of a multidisciplinary approach to rehabilitation for MFS [2, 6].

In 2014, we established the first inpatient rehabilitation program in Germany for people with MFS based on experiences from the National Resource Center for Rare Disorders in Norway. It is now offered annually to Marfan patients with a stable physical condition with the general objective to ensure or improve participation in all areas of life. The Marfan rehabilitation program lasts 3 weeks and is based on the standard German inpatient cardiac rehabilitation [7]. The main goals are to achieve the best possible support of patients’ capacities with respect to physical, psychological, and social aspects. Besides physicians, the rehabilitation team includes nursing staff, exercise therapists, physiotherapists, psychologists, nutritionists, and social workers.

We here present the results of an observational pilot study on the first two cohorts of patients with MFS, who underwent our newly designed rehabilitation program. The program was designed to meet the specific needs of young adults with MFS and similar syndromes, e.g. Loeys-Diez syndrome. These are predominantly characterized by uncertainties regarding physical fitness, body image, sexuality, and occupational abilities. Also, questions of how to deal with the inheritance of MFS and of how to inform others of their condition are central to MFS patients [3]. The primary aim of this study was to confirm that our rehabilitation program was feasible and medically save. Another aim of our study was to apply standardized instruments to assess the impact of our rehabilitation program on physical fitness and psychological wellbeing of participants.

## Methods

### Patients

We recruited patients for participation in the rehabilitation program by announcements through the German Marfan self-help organisation, by personal communication between patients and by the staff at the Clinic of Cardiology at the University Heart Centre, Hamburg. Inclusion criteria were: 1) diagnosis of MFS or similar syndrome (Q87.4 according to ICD-10) in stable condition proven by a MFS-specialist located in one of the Marfan-units in a German university clinic, 2) NYHA stadium < III, 3) time since last cardiovascular surgery > three months, 4) no relevant increase of aortic diameter within the last 12 months. Exclusion criteria were: severe psychiatric or other medical comorbidity. Two groups of patients comprising 8 individuals recruited in 2014 and 10 individuals recruited in 2015 completed the rehabilitation program in the Muehlenberg-Clinic - a rehabilitation clinic in Germany specialized in cardiac rehabilitation. Once patients were admitted to the rehabilitation clinic we offered participation in our pilot study. All patients gave their informed consent to participate. Sociodemographic data is presented in Table 1.

**Table 1:** Sociodemographic data

Variable	
Age	
m	46.7
sd	7.8
Years of school education	
m	10.8
sd	1,5
Gender (n)	
Female	14
Male	4
Professional Qualification (n)	
Blue collar	2
White collar	16
Marital Status (n)	
Single/no partner	1
Married	15
Divorced/Separated	2
Children (n)	
0	7
1	2
2	8
3	1

*m* mean, *sd* standard deviation, *n* number of patients

Mean age was 46.7 years, 71% were female. The educational level was relative high and most of the patients lived in stable relationships. 61% had children. Seventeen individuals were diagnosed as Marfan patients according to the Ghent criteria, and 1 patient was diagnosed as Loeys-Dietz Syndrome. Twelve patients had undergone operations of the aorta or the mitral valve not immediately prior to the rehabilitation.

The following psychiatric comorbidity according to ICD-10 was diagnosed in six patients: neurasthenia (F48.0): one patient, adjustment disorder (F43.2): two patients, insomnia (F51.0): one patient, agoraphobia (F40.0) and somatoform pain disorder (F45.41): one patient, recurrent depressive disorder (F33.0): one patient. Cardiovascular manifestations and type of related previous surgical and current pharmacological treatment is presented in Table 2.

**Table 2:** Cardiovascular manifestations and surgical / pharmacological treatment

Cardiovascular manifestations previously treated by surgery	Number of patients affected
mitral valve prolapse	3
tricuspid valve prolapse	1
aortic dissection stanford A	1
aortic dissection stanford B	2
aortic regurgitation	7
aortic aneurysma	9
mitral regurgitation	2
patent foramen ovale	1
pulmonary artery dilatation	1
Cardiovascular manifestations not yet treated by surgery	Number of patients affected
mild mitral valve prolapse	1
ascending aorta <45 mm	2
Corresponding medication	Number of patients treated with
angiotensin II receptor antagonist	13
beta 1 receptor blocker	8
aspirin	4
phenprocoumon	4
diuretics	1
calcium channel blocker	2
simvastatine	2
none (refused by patient)	2
Previous cardiovascular surgery	Number of patients treated with
david procedure	6
aortic valve replacement	4
mitral valve replacement	1
aortic prothesis	5
aortic valve reconstruction	2
mitral valve reconstruction	2
patent foramen ovale closure	1
Current NYHA functional classification	Number of patients
NYHA I	12
NYHA II	6

*NYHA* functional classification: New York Heart Association functional classification

### Rehabilitation program

After an extensive initial diagnostic work-up, we formulate the patients’ individual somatic, educative, psychological, and social rehabilitation goals. Based on these, we determine a rehabilitation plan, which is modified and adjusted to the success of rehabilitation. Our program is designed to admit individuals with MFS in groups of 10 persons at maximum. All individuals receive special education about Marfan disease such as talks by experts about current treatment options and behavior recommendations (6 h during the course of the program). Based on exercise electrocardiography performed at the beginning of the rehabilitation, we define the appropriate target heart rate for training (with a systolic blood pressure ≤ 160 mmHg as a stop criterion). Throughout the course of rehabilitation the training combines daily bicycle ergometry (30 min/day), gymnastics (4 × 60 min per week), fitness training (3 × 60 min per week) and nordic walking (3 × 60 min per week) adding up to at least three sport units per day. We provide patients with heart rate watches to independently monitor their heart rate during training sessions. The main goals here are to overcome patients’ uncertainty about their own physical abilities and, thereby, to improve patients’ sense of vitality. Twice a week (2 × 60 min per week), patients take part in a psychological group therapy focusing on coping with MFS. Additionally, patients are trained in progressive muscle relaxation according to Jacobson by a psychologist (2 × 30 min per week). The psychologist also offers one to one psychotherapeutic interventions according to demand (one hour per patient on average during the program). Counseling for job related issues (1 h per week) and for diet (1 h during the program) is offered and patients get introduced to a self-help organization for MFS (1 h during the program). As a core element of the rehabilitation process we review every patient in weekly multidisciplinary team conferences. Finally, an extensive examination is performed at the end of the rehabilitation to evaluate individual therapy success with regard to predefined therapy goals.

### Procedures

All patients answered the first set of psychometric questionnaires the day after admission to the rehabilitation program. Patients then took part in the three weeks rehabilitation program described above. The questionnaires for the final evaluation were handed out three days prior to discharge and collected the day before discharge. Physical fitness was recorded throughout the rehabilitation and documented in the medical records. At a 1 year follow-up, participants were postally contacted and asked to fill in the same set of psychometric questionnaires.

### Medical assessments

Medically adverse events that occurred during the rehabilitation were documented in the medical records of every participating patient according to clinical routine. Adverse events were defined as:

1. Any new cardiac arrhythmias such as atrial fibrillation, ventricular tachycardia, cardiac syncope
2. Any complications located at the aorta.

### Assessments of physical fitness

For all patients their maximum nordic walking distance as well as their maximum power in bicycle ergometry was continuously documented in the medical records. Maximum power in bicycle ergometry was defined as maximum Watt per kilogram bodyweight that patients were able to perform for at least 20 min with RR ≤ 160 mmHg. Fitness status regarding these two parameters was assessed within the first three days after admission and the last three days prior to discharge.

### Psychological assessments

Patients filled in the following questionnaires at the beginning and at the end of the rehabilitation program as well as one year after discharge from the program: The German version of the Hospital Anxiety and Depression Scale (HADS) ([8], German: [9]), Somatization subscale of the Symptom Checklist (SCL)-90-R ([10], German: [11]), Fatigue Severity Scale (FSS) ([12], German: [13]), Short-Form-36 (SF-36) ([14], German: [15]), Nottingham Health Profile (NHP) ([16], German: [17]).

The HADS is a 14 item scale. Seven of the items relate to anxiety and seven to depression. It is a widely used instrument for the assessment of anxiety and depression in patients in medical treatment settings. Its reliability and validity are well established. Suffering from diverse physical complaints was assessed by using the Somatization subscale of the SCL-90-R. The SCL is a very often used measure of psychological distress in clinical practice and research. A high number of studies have been conducted demonstrating the reliability, validity and utility of the instrument. The FSS is a short (9 items) unidimensional instrument for an economic assessment of the severity of fatigue in different situations during the past week in groups of patients with somatic disorders. It is able to differentiate patients with fatigue from healthy controls with good internal consistency and test-retest-reliability. The SF-36 was derived from the General Health Survey of the Medical Outcomes Study [14]. It is one of the most widely used generic measures of health-related quality of life and has shown to discriminate between subjects with different chronic conditions and between subjects with different severity levels of the same disease. The SF-36 has also demonstrated sensitivity to significant treatment effects in a variety of patient populations. The SF-36 generates 8 subscales. The 8 subscales are: physical functioning, physical role functioning, bodily pain, general health perceptions, vitality, social role functioning, emotional role functioning, and mental health. The SF-36 yields two summary scores: physical component summary (PCS-36) and mental component summary (MCS-36). They are calculated and linearly transformed with respect to the US norm population. The NHP is a generic quality of life instrument that was developed to be used in epidemiological studies of health and disease. Part I contains 38 yes/no items in 6 dimensions: pain, physical mobility, emotional reaction, energy, social isolation and sleep. Part II contains 7 general yes/no questions concerning daily living problems. The two parts may be used independently and part II is not analysed in this study. Part I is scored using weighted values which give a range of possible scores from zero (no problems at all) to 100 (presence of all problems within a dimension). The NHP scale has proved capable of measuring changes in perceived health following different treatments such as surgery and rehabilitation.

### Analyses of data

Data were analysed by calculating t-tests for repeated measures from admission to discharge for each of the scales and parameters mentioned above in order to evaluate rehabilitation success. Additionally, admission to discharge-effect-sizes (Cohen’s *d*) were computed (0.2–0.5: small effect; 0.5–0.8: medium effect; > 0.8: large effect). Admission to follow-up t-tests and effect sizes were calculated for the psychological assessments. For all scales positive effect sizes indicate an improvement over time.

## Results

No adverse medical event was reported for any of the patients during the time of the rehabilitation.

### Physical fitness

Physical fitness improved from admission to discharge for both assessment parameters (maximum power in bicycle ergometry for at least 20 min, *p* < .001, and maximum nordic walking distance, *p* < .05). Statistical significance is confirmed by large effect sizes (Table 3).

**Table 3:** Analyses of admission to discharge differences in assessments of physical fitness by t-tests and effect-sizes (Cohen’s *d*)

	Admission		Discharge		Admission to discharge			
Assessment parameter	m	sd	m	sd	n^a^	t	p	Cohen’s *d*
Maximum Watt per kg body weight for at least 20 min	.54	.21	.77	.23	17	4.455	<.001	1.1
Maximum nordic walking distance in meter	1694	893	2356	666	17	3.492	<.05	0.8

*m* mean, *sd* standard deviation, *n* sample size, t-value in t-test for repeated measurements; *p* level of statistical significance, *Cohen’s d*: effect size of pre-post-differences

^a^Data from 1 patient of the first cohort is missing

### Psychological assessments

#### Admission to discharge differences

Psychological distress decreased according to the HADS (anxiety and depression subscales) and NHP (emotional reaction) and in terms of somatization (SCL-90-R) and fatigue/loss of energy (FSS; NHP) (*p* < .05, Table 4). Differences (*p* < .001) between pre-post examinations were also found for the vitality and the mental health subscales as well as for the mental health summary scale of the SF-36. Impairments due to perception of pain decreased (*p* < .05) according to SF-36 (bodily pain, Table 4). These statistically significant pre-post-differences were confirmed by small (SF-36 bodily pain) and medium (HADS anxiety and depression, NHP emotional reaction, SCL-90-R somatization, FSS, NHP energy, and MCS-36) to large (SF- 36 vitality and mental health) pre-post effect sizes.

**Table 4:** Analyses of admission-discharge and admission-follow-up differences in psychological assessments by t-tests for repeated measurements and effect-sizes (Cohen’s *d*)

assessment scale	admission			discharge			admission to discharge			follow-up			admission to follow-up		
	m	sd	n	m	sd	n^a^	T	p	*d*	M	sd	n^b^	T	p	*d*
HADS Depression	7.17	4.69	18	5.06	4.21	18	3.43	<.05	0.5	5.0	4.47	16	2.38	<.05	0.5
HADS Anxiety	9.61	5.26	18	6.67	5.42	18	4.17	<.05	0.6	7.0	4.32	16	2.33	<.05	0.5
SCL-90-R Somatization	23.1	7.80	18	20.6	6.36	18	2.14	<.05	0.4	22.3	7.74	16	−.1	.922	0.1
FSS	5.62	.91	18	5.14	1.10	18	2.45	<.05	0.5	4,92	1.51	16	2.33	<.05	0.6
SF-36 physical functioning	59.4	18.1	18	65.6	21.96	18	−1.96	.067	0.3	67.7	23.7	15	−1.61	.129	0.4
SF-36 physical role functioning	41.7	40.2	18	58.3	40.22	18	−1.65	.117	0.4	66,7	40.8	15	−3.29	<.05	0.6
SF-36 bodily pain	46.1	28.8	18	58.4	28.97	17	−2.31	<.05	0.4	51.2	25.7	15	−1.14	.272	0.2
SF-36 general health perception	41.3	15.4	18	47.1	15.52	18	−1.81	.089	0.4	48,6	19.0	14	−1.52	.154	0.4
SF-36 vitality	34.7	13.9	18	51.4	18.13	18	−5.56	<.001	1.0	42.3	21.0	15	−1.79	.096	0.4
SF-36 social role functioning	67.4	33.3	18	74.3	25.18	17	−1.16	.264	0.2	77.7	27.4	14	−1.38	.192	0.3
SF-36 emotional role limitation	59.3	45.1	18	74.5	40.02	17	−1.94	.070	0.4	75.6	38.8	15	−1.42	.178	0.4
SF-36 mental health	59.3	20.7	18	73.8	17.26	18	−4.84	<.001	0.8	66.9	20.5	14	−1.96	.071	0.4
PCS-36	36.3	8.64	18	38.7	9.7	17	−1.22	.240	0.3	40.6	10.4	14	−2.02	.064	0.5
MCS-36	43.4	13.3	18	50.1	10.5	17	−4.13	<.001	0.6	47.8	11.5	14	−1.59	.135	0.4
NHP pain	30.6	33.0	18	25.0	32.65	18	.95	.354	0.2	21,7	31.9	15	.96	.352	0.3
NHP physical mobility	19.4	16.2	18	16.7	19.17	18	.89	.386	0.2	11.7	18.0	15	1.52	.150	0.5
NHP emotional reaction	28.4	26.7	18	14.8	24.10	18	3.42	<.05	0.5	14.1	19.0	15	2.29	<.05	0.6
NHP energy	48.2	40.0	18	29.6	35.95	18	2.15	<.05	0.5	33.3	35.7	15	1.52	.150	0.4
NHP social isolation	8.89	17.1	18	10.0	14.14	18	−.56	.579	−0.1	13.3	19.5	15	−.81	.433	−0.2
NHP sleep	30.0	30.9	18	23.3	28.49	18	2.06	.055	0.2	21.3	29.7	14	.52	.610	0.3

*M* mean, *sd* standard deviation, *n* sample size, *T* t-value in t-test for repeated measurements, *p* level of statistical significance, *d* Cohen’s *d*, *HADS* Hospital anxiety and depression Scale, *SCL-90-R* Symptom Checklist-90-Revised, *FSS* Fatigue Severity Scale, *SF-36* Short Form 36, *PCS-36* physical health summary scale of SF-36, *MCS-36* mental health summary scale of SF-36, *NHP* Nottingham Health Profile

^a^Data from 1 patient of the first cohort is missing

^b^Data from up to 4 patients is missing

#### Admission to follow-up differences

**Fig. 1:**
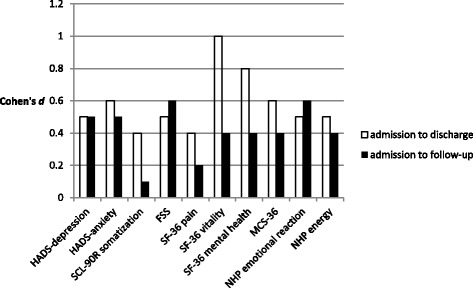
Admission-discharge and admission-follow-up effect sizes for psychological assessments with statistically significant admission-discharge improvements. Cohen’s *d*: effect size; HADS: Hospital anxiety and depression Scale; SCL-90-R: Symptom Checklist-90-Revised; FSS: Fatigue Severity Scale; SF-36: Short Form 36; MCS-36: mental health summary scale of SF-36; NHP: Nottingham Health Profile

16 out of 18 patients who were contacted for the one year psychological follow-up assessment returned their questionnaires. Some patients did not completely fill out all questionnaires. Significant differences were found for psychological distress (HADS anxiety and depression subscales), somatization (SCL-90-R), for SF-36 physical role functioning, and for NHP emotional reaction. Admission to discharge effects mostly persisted through the one year follow-up but declined to smaller sizes (Table 4 and Fig. 1).

## Discussion

Inpatient rehabilitation with daily exercise and with an emphasis on endurance training seems to be safe and helpful for individuals with MFS. The 160 mmHg stop criterion for defining the target heart rate proofed to be a useful tool and an easily manageable procedure throughout the whole course of the rehabilitation. Although the moderate target heart rate limits physical stress during training, it nevertheless allowed for adequate and sufficient training effects. Training under these conditions seems to be effective for patients with MFS.

There are no randomized controlled studies to demonstrate the efficacy of psychological interventions on depression in patients with congenital heart disease [18]. Conversely, physical exercise regimens are well-known to reduce depression and anxiety in various clinical settings [19]. Using the HADS as a screening tool for depression and anxiety, two cut-off scores have generally been used in most studies: scores of 8 to 10 corresponding to doubtful cases and scores of 11 and higher identifying valid cases [20, 21]. In our sample, even though six patients had psychiatric comorbidity, the medium anxiety scores of MFS patients at admission were within the range of doubtful cases and depression scores were within normal range. Similar results have been reported in other studies on MFS-patients [3]. In the study reported here, both depression and anxiety decreased significantly during the course of rehabilitation. This result is in line with findings in other clinical samples [19]. We also found improvements regarding somatization (SCL-90-R) and fatigue (FSS), suggesting that not only anxiety and depression but also psychological wellbeing in general improved. Fatigue at admission was similar to the level of fatigue reported by Bathen et al. [5] for a Norwegian sample of MFS patients.

It might be considered unrealistic to expect participating in a three weeks rehabilitation program to influence quality of life for patients with MFS because quality of life is a broad and comprehensive psychological construct. Interestingly, our rather short three week program was, in fact, sufficient to increase quality of life in MFS patients. SF-36 MCS- and PCS-scores at admission were comparable with data from other studies with MFS patients [4, 22] and indicate a reduced quality of life in comparison to normal control subjects and patients with coronary heart disease [23]. The same result was found for the NHP scores at admission. They were higher, indicating lower quality of life than measured, for example, in a sample of survivors of myocardial infarction [24]. This was not true for the social isolation (SI) score. SI was low signifying a low level of interactional problems for the MFS patients. All SF-36 and NHP subscales indicated less distress at discharge than at admission except for the SI-score which was slightly higher at discharge without being statistically significant. Statistically significant changes were found for physical pain (SF-36), mental health (SF-36 subscale and summary scale), emotional reaction (NHP) and energy (NHP). The largest effect size was found in regards to vitality (SF-36). This might be due to the fact that overcoming a sense of uncertainty regarding physical abilities in a peer group of patients with the same diagnosis could help improving a body-related sense of well being [25]. These positive changes in psychological functioning throughout the course of rehabilitation point toward a positive effect of this specific treatment not only on physical variables but also on quality of life in general. Similarly, cardiac rehabilitation is known to have a positive influence on quality of life in patients with coronary heart disease [26] and exercise interventions seem to have a positive effect on quality of life in clinical and in non-clinical populations [27]. This is especially valuable because quality of life is known to be related to many different and important aspects of human life, such as, for instance, physical health, family and education [28].

Contrastingly, the changes in SF-36 physical health summary scale as well as the SF-36 physical mobility, SF-36 physical functioning and SF-36 general health perception did not reach statistical significance and corresponding effect sizes were small. The objective improvements in physical fitness (Table 3) did not translate fully into a better perception of physical wellbeing. This might be due to the chronicity of MFS. Three weeks of training, even though they may result in a substantial increase in physical fitness, might not be enough to change the mental representation of an individual’s physical abilities when the person has grown accustomed to think of her- or himself as handicapped by a chronic condition.

Particularly promising are the follow-up improvements in psychological assessments. Generally, research in cardiac rehabilitation suggests that maintaining improvements throughout follow-up is much more difficult than proving effectiveness while participants are still engaged in the rehabilitation program [7]. In our study, we observed stable and significant reductions in psychological distress (depression, anxiety, fatigue, emotional reaction) with medium effect sizes, not only during attendance of the rehabilitation program but also over the one year follow-up time. For improvements in vitality and mental health, we still found small, albeit non-significant effects after one year, whereas initial effects were large. Physical role functioning further increased significantly from admission to follow-up although there was just a small effect of rehabilitation on physical role functioning from admission to discharge.

We did not expect our three-week intervention to reliably affect psychological wellbeing over a one year follow-up period. In our small sample, we nevertheless found some persistent positive changes not primarily in quality of life, but in core psychological functioning, i.e. depression, anxiety, and fatigue. All three variables are known to be closely related [29]. Also, the emotional reaction scale of the NHP indicates positive changes in the area of psychological functioning beyond the original duration of the three-week rehabilitation program. However, statistical significance of some admission-to-discharge improvements in psychological functioning such as somatization, SF-36 bodily pain, SF-36 vitality, SF-36 mental health, SF-36 MCS, and NHP energy were not sustained until follow-up. Perception of pain is known to be a main problem in MFS and is associated with disability and psychological strain [3, 22]. Therefore, it is particularly dissatisfying that perception of pain recurred at follow-up.

Generally, our sample size was small and because of missing data at follow-up, the statistical power of comparisons between admission and follow-up was even more limited and statistical significant differences could hardly be detected. It is currently uncertain whether the results obtained in this sample of well-educated patients living in stable relationships can be generalized to the whole population of MFS patients. Also, psychological distress at admission was low in terms of anxiety and depression which makes it difficult to demonstrate clinically meaningful improvement. On the other hand, patients were likely highly motivated to benefit from participating in the program because it is a new opportunity for MFS patients in Germany. Nevertheless, taking part in the rehabilitation program might have had some positive impact on psychological wellbeing not only for a short period of time but also on a long term basis. However, without a control group we cannot unequivocally attribute the documented changes to the treatment. Therefore, a controlled design with randomized assignment to either treatment or control group is needed.

### Study limits

The results presented here have some limitations. Due to the small number of participants the power of the study is limited. The study was not controlled, which means that the pre-post-differences cannot unequivocally be attributed to the treatment. So far, only one-year follow-up data are available. Therefore, long-term effects of the rehabilitation program remain unknown. Although questionnaire data from 16 out of 18 patients at follow-up is sufficient to draw meaningful conclusions we still do not know what happened to the two participants who did not provide us with their follow-up data. The treatment is a comprehensive and multidisciplinary approach which makes it difficult to identify specifically effective treatment components. Physical activity and psychological wellbeing are known to be closely related [19]. Physical activity contributes to all aspects of quality of life, i.e. physical, psychological and social. Exercise proved to have positive effects mainly in clinical but also in non-clinical populations. Nevertheless, the direction of causality remains unclear and psychosocial benefits may also occur in the absence of clearly identifiable changes in physiological parameters, just as it is possible to establish physiological changes in the absence of any perceived psychological benefits. The participants are mainly well-educated, occupy rather high professional positions and live in stable relationships. Future studies should investigate whether our treatment proves useful for participants from diverse socio-demographic and socio-economic backgrounds.

## Conclusions

Overall, our three week rehabilitation program specific designed for individuals with MFS proved useful in order to improve physical fitness and psychological wellbeing. Patients were highly satisfied with the programme and improved in almost all psychological and physical fitness assessments. Considerable improvements persisted through a one-year follow-up period. Medical assessments ruled out medical problems or adverse events caused by participation in the programme. For the future, an evaluation of the efficacy of the programme in a controlled design including follow-up assessments as well as further conceptual improvements on the basis of existing experience is desirable. The results should also be used to develop similar programmes for patients with other congenital heart defects.

## Abbreviations

FSS: Fatigue Severity Scale
HADS: Hospital Anxiety and Depression Scale
MFS: Marfan syndrome
NHP: Nottingham Health Profile
SCL: Symptom Checklist
SF-36: Short Form-36
